# Home physiotherapy with *vs*. without supervision of physiotherapist for assessing manipulation under anaesthesia after total knee arthroplasty

**DOI:** 10.1186/s42836-020-00063-3

**Published:** 2021-03-03

**Authors:** Sanjay Bhalchandra Londhe, Ravi Vinod Shah, Amit Pankaj Doshi, Shubhankar Sanjay Londhe, Kavita Subhedar, Krishnan Iyengar, Prashant Mukkannavar

**Affiliations:** 1 Orthopaedic surgeon, Criticare Hospital, Plot No 516, Besides SBI, Teli Gali, Andheri East, Mumbai, Maharashtra 400069 India; 2 Orthopaedic Surgeon, Hinduja Healthcare, Khar, India; 3 Meril Life Sciences, Mumbai, India; 4 Dr Vishwanath Karad MIT World Peace University, Pune, India; 5 Criticare Hospital, Mumbai, India; 6 Alpha speciality Clinic, Mumbai, India; 7 SDM College of Physiotherapy, Sattur, Dharwad, Karnataka India

**Keywords:** Total knee arthroplasty, Manipulation under anaesthesia, Postoperative physiotherapy, Stiff knee

## Abstract

**Abstract:**

The aim of this retrospective cohort study was to compare home physiotherapy with or without supervision of physiotherapist for assessing manipulation under anaesthesia after total knee arthroplasty.

**Methods:**

A total of 900 patients (including 810 females and 90 males) who had undergone total knee arthroplasty were divided into group A (*n* = 300) and group B (*n* = 600). Patients in group A had home physiotherapy on their own after discharge from hospital. The physiotherapist did not visit them at home. Patients in group B received home physiotherapy under supervision of physiotherapist for 6 weeks after discharge from hospital. Patients’ age, range of motion of the knee, and forgotten joint score-12 were assessed. A *p* < 0.05 was considered statistically significant.

**Results:**

In group A, the mean age was 69.1 ± 14.3 years (range: 58 to 82 years); in group B, the mean age was 66.5 ± 15.7 years (range: 56 to 83 years) (*p* > 0.05). Preoperatively, the mean range of motion of the knee in group A and B was 95.8° ± 18.1° and 95.4° ± 17.8°, respectively (*p* > 0.05). The mean forgotten joint score-12 of group A and B were 11.90 ± 11.3 and 11.72 ± 12.1 (*p* > 0.05), respectively. Six weeks after total knee arthroplasty, the mean ROM of the knee in group A and B was 109.7° ± 22.3° and 121° ± 21.5°, respectively (*p* < 0.05). The mean postoperative forgotten joint score-12 of the group A and B was 24.5 ± 16.4 and 25.6 ± 17.4, respectively (*p* > 0.05). The rate of manipulation under anaesthesia was 3% in group A and 0.2% in group B (*p* < 0.05).

**Conclusion:**

After total knee arthroplasty, frequent physiotherapist’s instruction helps the patients improve knee exercises and therefore decrease the risk of revision surgery. The home physiotherapy under supervision of physiotherapist lowers the rate of manipulation under anaesthesia.

**Level of evidence:**

Therapeutic study, Level IIa.

## Introduction

Total Knee Arthroplasty (TKA) is one of the most successful operations in modern orthopaedics [1]. TKA is highly effective in relieving pain and improving functions, including activities of daily living [2–6]. Huge advances have been made in the implant designs, refinement of the surgical treatment of TKA and patient selection. Bourne *et al*. [7] and other authors showed that a sizable percentage of patients, *i.e*., 19%. remain dissatisfied with their primary TKA [8–13]. One of the common reasons in these 19% unsatisfied patients is development of knee stiffness after the TKA surgery. If untreated, knee stiffness may progress over time and affects patients’ ability to perform daily tasks [14]. Early gait analyses and biomechanical studies showed that the knee should achieve at least 83° of flexion to ascend stairs, 90° to 100° of flexion to descend stairs, 93° to 105° flexion to rise from a chair, and more than 120° of flexion to squat or kneel [15–17]. Till now, there is no clear consensus on the definition of knee stiffness in the literature. Usually, a stiff knee is confirmed when the knee flexed less than 90° after TKA [18–20]. Some factors may be associated with knee stiffness, including a decreased preoperative ROM, younger age, diabetes mellitus, socio-economic status, and previous knee surgery, malpositioning of implant, inadequate resection, and over-stuffing of the component [21–24].

The objective of this retrospective cohort study was to compare home physiotherapy with and without supervision of physiotherapist for assessing MUA after TKA. We also reported the efficacy of postoperative home physiotherapy under supervision of physiotherapist.

## Patients and methods

The institutional review boards of the participating hospitals approved the study. Informed consent was obtained from each patient.

A total of 900 patients (involving 810 females and 90 males) were included in this study and divided into two groups. Group A consisted of 300 patients who had undergone TKA between January 2011 and December 2013; group B included 600 patients who had undergone TKA between January 2014 and December 2018.

Preoperatively, we recorded patients’ age, body mass index (BMI), ROM of the knee, diagnosis, degree of varus deformity, 100-mm visual analogue score (VAS) for knee pain, and forgotten joint score (FJS)-12 (Table 1). The duration of hospital stay, and hip-knee-ankle alignment were also recorded (Table 2). An independent observer who did not attend the treatments assessed all the data. MUA was carried out when the knee flexion was less than 90° at the end of the sixth postoperative week. The study was started with null hypothesis. Using a pocket goniometer, ROM (angle of maximal flexion to extension) of the knee was measured by an independent observer (nurse practitioner). Preoperative data were recorded 1 week prior to TKA.

**Table 1:** Comparison of preoperative characteristics of patients between Group A and Group B

Parameters	Group A	Group B	***p*** value
**Number of patients **(*n*)	300	600	**–**
**Mean Age (years)**	69.1 ± 14.3 (58–82)	66.5 ± 15.7 (56–83)	0.5780
**Mean BMI (kg/m**^**2**^**)**	28.2 ± 4.7	27.6 ± 5.4	0.1016
**Gender**			
Females	*n* = 269 (89.66%)	*n* = 542 (90.33%)	0.7511
Males	*n* = 31 (10.33%)	*n* = 58 (9.66%)	0.7510
**Mean Preoperative ROM**	95.8 ± 18.1	95.4 ± 17.8	0.7521
**Preoperative degree of deformity (varus)**	8.5 ± 2.6	9.8 ± 3.1	0.1498
**Preoperative clinical diagnosis**			
OA	*n* = 268 (89.33%)	*n* = 533 (88.83%)	0.8213
RA	*n* = 30 (10%)	*n* = 62 (10.33%)	0.8776
Others	*n* = 2 (0.67%)	*n* = 5 (0.83%)	0.7967
**Preoperative Associated co-morbidities:**			
Cardiac	112 (37.33)	230 (38.33)	0.7709
Renal	53 (17.66)	137 (22.833)	0.0732
Hepatic	10 (3.33)	16 (2.833)	0.6594
**Mean Preoperative FJS-12**	11.90 ± 11.3	11.72 ± 12.1	0.8298
**Mean Preoperative VAS score**	7.4 ± 2.9	7.5 ± 1.5	0.3899

**Table 2:** Comparison of postoperative characteristics of patients between Group A and Group B

Parameters	Group A	Group B	***p*** value
**Mean hospital stay duration (days)**	4.4 ± 0.5	4.6 ± 0.4	0.0734
**Mean Postoperative VAS score at 6 weeks**	4.5 ± 1.8	4.3 ± 1.4	0.0674
**Mean Postoperative FJS-12 at 6 weeks**	24.5 ± 16.4	25.6 ± 17.4	0.3625
**Mean Postoperative Knee Alignment (HKA angle or FT angle) (degrees)**	181.4 ± 0.5	182.5 ± 0.2	0.1302
**Mean Postoperative ROM**	109.7 ± 22.3	121.3 ± 21.5	< 0.0001
**MUA Rate**	*n* = 9 (3%)	*n* = 1 (0.1667%)	0.0001

### TKA and physiotherapy

All operations were done by the same surgical team. Operation was performed under tourniquet control and through the standard parapatellar approach. We used a cemented posterior stabilized knee implant (Freedom Total Knee system, Maxx Orthopedics Inc., PA, USA) in all patients. The physiotherapy was started immediately after TKA, *i.e.*, on the very same day of the operation. The physiotherapy was in the form of muscle strengthening exercises, ROM exercises, closed chain exercises, and practice of stair climbing and gait training. The muscle strengthening exercises were in the form of ankle pump, static and dynamic quadriceps muscle strengthening, and glutei and hamstring muscle strengthening exercises. The ROM exercises comprised of passive, assisted active and active knee movements. Gait training included walking initially with walking aid like walker and gradually progressing to walking stick.

Patients of group A were instructed to have physiotherapy on their own at home. The physiotherapist did not visit them at home. The physiotherapist just gave them instructions at the time of discharge from the hospital. We provided information booklets to show how the physiotherapy exercises were performed.

Patients of group B received postoperative physiotherapy at home with physiotherapist visiting their home for 6 weeks. The physiotherapist visited the patients at home initially 6 days a week (excluding Sunday) for the first 2 weeks, 3 days a week for the next 2 weeks, and then once a week for the final 2 weeks. The same physiotherapy team conducted the home physiotherapy. The information conveyed by the physiotherapist included ROM, walking with/without walking aid, ability to perform day to day activities, and amount of knee pain the patient was experiencing.

MUA was applied when the patients had failed to achieve 90° of ROM at the first follow-up visit (6 weeks after TKA). Under general anaesthesia, the patient underwent MUA as a day care procedure and was put on standard post-TKA rehabilitation program. Postoperative data were recorded at each clinic visit.

### Statistical analysis

Data were expressed as mean ± standard deviation. Comparisons between the groups were made by using Student’s unpaired *t*-test. The null hypothesis was tested with chi-squared test and *t*-test. A *p* < 0.05 was considered statistically significant.

## Results

The results were expressed as the mean ± standard deviation. In group A, the mean age was 69.1 ± 14.3 years (range: 58 to 82 years). In group B, the mean age was 66.5 ± 15.7 years (range: 56 to 83 years). Preoperatively, the mean ROM of the knee in group A and B were 95.8° ± 18.1° and 95.4° ± 17.8°, respectively (*p* = 0.7521). The mean varus of deformity in group A and group B were 8.5° ± 2.6° and 9.8 ± 3.1°, respectively (*p* = 0.1498). The mean FJS-12 scores of group A and B were 11.90 ± 11.3 and 11.72 ± 12.1 (*p* = 0.8298). The mean hospital stay for patients in group A and B was 4.4 ± 0.5 and 4.6 ± 0.4 days, respectively (Table 1).

Six weeks after TKA, the mean ROM of the knee in group A and B was 109.7° ± 22.3° and 121° ± 21.5°, respectively (*p* < 0.0001). Postoperative hip-knee-ankle angle of the group A and B was 181.4° ± 0.5° and 182.5° ± 0.2°, respectively (*p* = 0.1302). The mean postoperative FJS-12 scores of the group A and B were 24.5 ± 16.4 and 25.6 ± 17.4, respectively (*p* = 0.3625). The MUA rates were 3% in group A and 0.2% in group B (*p* = 0.0001) (Table 2).

The sample size was estimated to be 870 with ɑ error of 0.05, β error of 0.1, and power of 90. Considering dropouts, it was rounded to 900 patients who had undergone TKA. The MUA rate of group B (0.2%) was less than that of group A (3%) (*p* = 0.0001). The MUA rate of group B was also less than the MUA rates (4 to 6%) reported in large Western cohorts (*p* < 0.001; 95% confidence interval − 0.002) (Table 3).

**Table 3:** MUA rate of current study *versus* the rates of other large cohorts with *p* values

Author	Country of study	Patients (*n*)	MUA rate	***p*** value
Werner	USA	141016	4.3	< 0.001
Bawa	USA	3224	4.3	< 0.001
Issa	USA	3128	4.9	< 0.001
Pamilo	Finland	624	5.9	< 0.001
Wied	Denmark	259	5.8	< 0.001
Current study	India	600	0.167	

## Discussion

Stiff knee after primary TKA is a very debilitating condition. If left untreated, it will affect patients’ daily activities, such as climbing up and down the stairs, rising from a chair, or tying shoelaces that routinely requires the knee to flex more than 90° [17, 25–27]. Asian population even requires more than 120° to perform certain activities, such as squatting and sitting cross-legged.

We found that physiotherapy at home with physiotherapist visiting decreased the MUA rate, compared with no visiting. Our results were also lower than those of the previous large cohort studies. We believe the constant feedback mechanism between the physiotherapists and patients is helpful in reducing the incidence of stiff knee, thereby decreasing the MUA rate.

Many treatment options are available for managing the stiff knee. Initially, the patients are subjected to aggressive physiotherapy. If the physiotherapy fails to help the patient achieve an acceptable ROM, MUA is indicated. Even though the MUA helps in achieving satisfactory ROM after the procedure, the occurrence of stiff knee should be prevented in the first place. Werner *et al*. [28] showed that MUA applied within 6 months after TKA increased the risk of early revision of TKA.

Some factors may be associated with the development of stiff knee after TKA. Werner *et al*. [28] found age < 65 years, female gender, and smoking were associated with a high MUA rate. Issa *et al*. [29] showed that white race, preoperative diabetes, high cholesterol levels, preoperative ROM < 100°, and osteonecrosis of the knee were associated with an increased MUA rate.

The study had several limitations. First, this study was not a prospective, blinded, or randomized study. Patient characteristics, such as age, BMI, ROM, varus deformity, knee pain, and FJS-12 may not reflect the actual statistical differences. The cost of both types of physiotherapies was not assessed, which might affect patients’ selection of treatments.

## Conclusion

After TKA, frequent physiotherapist’s instruction helps the patients improve knee exercises and therefore decrease the risk of revision surgery. The home physiotherapy under supervision of physiotherapist also decreases the MUA rate.

## Data Availability

Not Applicable.

## Abbreviations

TKA: Total Knee Arthroplasty
MUA: Manipulation Under Anaesthesia
ROM: Range of Motion
VAS: Visual Analogue Score
FJS-12: Forgotten Joint Score-12
